# Ketamine for prehospital trauma analgesia in a low-resource rural trauma system: a retrospective comparative study of ketamine and opioid analgesia in a ten-year cohort in Iraq

**DOI:** 10.1186/s13049-015-0176-1

**Published:** 2015-11-09

**Authors:** Ole Kristian Losvik, Mudhafar Kareem Murad, Eystein Skjerve, Hans Husum

**Affiliations:** Department of Clinical Medicine, Faculty of Health Sciences, University of Tromso, PO Box 6050 Langnes, Tromso, 9037 Norway; Trauma Care Foundation Iraq, Sulaymaniyah, Iraq; Norwegian University of Life Sciences, Ås, Norway; Tromso Mine Victim Resource Centre, University Hospital of North Norway, PO Box 80, Tromso, 9038 Norway

**Keywords:** Analgesia, Ketamine, Pain relief, Prehospital, Prehospital analgesia, Trauma, War

## Abstract

**Background:**

Opioid analgesics are used in most trauma systems, and only a few studies report on the use of ketamine for prehospital analgesia. In a low-cost rural trauma system in Iraq paramedics have been using prehospital ketamine analgesia for ten years. This study aims to evaluate the effects of prehospital analgesia on physiologic trauma severity indicators and compare the effect of ketamine and pentazocine on those indicators.

**Methods:**

The investigation was conducted as a retrospective cohort study with parallel group design. Three subsamples of trauma patients were compared: no analgesia (*n* = 275), pentazocine analgesia (*n* = 888), and ketamine analgesia (*n* = 713). Physiologic severity scores were calculated based on rated values for respiratory rate, blood pressure, and consciousness. The associations between outcomes and explanatory variables were assessed using a generalized linear model.

**Results:**

Paramedic administration of analgesia was associated with a better physiologic severity score (PSS) outcome (*p* = 0.01). In the two subsamples receiving analgesia significantly better outcomes were observed for respiration (*p* < 0.0001) and systolic blood pressure (*p* < 0.0001). In patients with Injury Severity Score >8 ketamine was associated with a significantly better effect on the systolic blood pressure compared to opioid analgesia (*p* = 0.03).

**Conclusion:**

Prehospital analgesia for trauma victims improves physiologic severity indicators in a low-resource trauma system. Compared to pentazocine, ketamine was associated with improved blood pressure for patients with serious injuries. In a low-resource setting, ketamine seems to be a good choice for prehospital analgesia in trauma patients.

## Background

In addition to the humane reasons for providing optimal pain relief, pain relief is required because the systemic response to pain affects nearly all organ systems. Recent changes in trauma care include an emphasis on pain treatment to decrease the potent inflammatory response that results in hyper-coagulability, organ dysfunction, systemic inflammatory response, lung injury, brain injury, depression, and post-traumatic stress disorder [1].

In most prehospital trauma systems, opioid analgesics have been the preferred choice over the last decades. However, the therapeutic range is narrow in opioids; there is the risk of accidental overdose that can cause respiratory depression, hypotension and the loss of protective airway reflexes.

Ketamine hydrochloride is a non-opioid potent analgesic used for anesthesia for decades. Ketamine stimulates the sympathetic nervous system and moderately increases heart rate and systolic blood pressure; these side effects might benefit trauma victims. Ketamine does not affect respiration or laryngeal reflexes; under ketamine analgesia and anesthesia, patients breathe spontaneously and maintain airway control [2].

Hence, the drug does not have the same dangerous side effects as opioids, even if accidentally administered in excessive doses [3, 4]. Side effects reported include dysphoria, agitation, disorientation, felling unreality, nausea and vomiting are reported. Few studies report on the prevalence of side effects, however some information is to be found in one prospective study comparing ketamine analgesia to morphine in Vietnam. This study reported an analgesic effect similar to morphine, but with significant lower risk of vomiting [5]. In general, there are few scientific studies on the prehospital use of analgesics [6], and the under-treatment of pain is commonly reported [7].

The London helicopter emergency medical service has reported on their use of ketamine for analgesia and procedural sedation and concluded that ketamine is safe when used by physicians in prehospital trauma care [8]. To the best of our knowledge, no larger studies have been published on the paramedic use of ketamine for prehospital analgesia.

Some studies have looked into the addition of ketamine to morphine analgesia. In a study including 135 patients receiving either morphine alone or with addition of ketamine, the combination were shown to be superior. However, there were more adverse effects reported, most commonly disorientation reported for 11 % of the patients [9]. A similar prevalence of agitation was found in a study in Vietnam [5]. A Swedish randomized study including 26 patients, and found that the addition of ketamine to opioids provided safe analgesia without significant side effects [10].

In a low-cost rural prehospital trauma system, we aimed to assess the effects of the paramedic administration of prehospital analgesia on physiologic trauma severity indicators and compare the effect of prehospital ketamine and pentazocine analgesia on physiologic severity indicators.

## Methods

### Study design

The study was conducted as a non-randomized retrospective cohort study with a parallel group design. The patients were analysed in three subset based on prehospital analgesia: no analgesia, pentazocine analgesia or ketamine analgesia. The reference population for the study are trauma patients managed by rural, low-cost trauma systems.

### Study setting and trauma system

The intervention was conducted in the warzones and minefields of North and Central Iraq. The trauma system was initiated in 1997 to rescue land mine victims from the vast mine fields in the mountainous areas along the Iran-Iraq border, and it was later expanded to the Central Iraq war zone. The prehospital trauma system under study is a three-tiered system that consists of lay first responders (“first helpers”), trained paramedics at local clinics and district hospitals, and emergency room staff at the surgical hospitals.

Evacuations are often rough and difficult for both rural trauma scenarios and urban mass casualties; patients may be carried off-road without ambulances, and the first helpers may not be able to provide continuous close care and monitoring of vital signs.

The training began in 1997 when 20 paramedics were trained at rural health centres and equipped for advanced trauma life support by two of the authors (MKM, HH). The training consisted of three 150-h courses over a 3-year period [11]. Details on the system expansion and maturation have been previously reported [12]. By the end of 2006, the entire trauma system consisted of 135 paramedics and more than 7000 layperson first helpers were supervised by six local medical doctors.

### Participants

The study population comprised adult trauma patients who were consecutively managed by the trauma system from January 1997 to December 2006. The physiological responses to trauma differ between children and adults; therefore, trauma patients aged <15 years were not included in the study but will be reported in a separate study.

### Treatment protocol

The first dose of analgesics was administered at the scene as soon as the initial assessment of vital functions was assessed and intravenous access was established. Due to local medical traditions and the lack of evidence-based studies of ketamine analgesia, pentazocine was mainly used in blunt injuries and all cases of eye and/or traumatic brain injury. The drug was administered IV with an initial dosage of 20–30 mg for adults (0.4 mg/kg). Ketamine was used in all other cases of penetrating injuries and burns, with an initial IV dose of 0.2 mg/kg as a separate injection. In cases of agitation and unrest, diazepam (*n* = 228) was co-administered to the ketamine patients in one single IV dose of 5 mg. During protracted evacuations with repeated ketamine injections, 1 mg atropine (*n* = 63) was also administered IV to prevent excessive salivation. For both analgesics, repeated doses were given for clinical indications during assisted evacuations. Unfortunately, the trauma registry does not contain information on the dosage or frequency of drug administration.

### Outcome measures

The main outcome variable was the change in the physiologic severity score (PSS) [13]. The PSS was calculated from blood pressure, respiratory rate and consciousness level measurements in a component system similar to the Revised Trauma Score for Triage (RTS-Triage). The PSS uses a simplified consciousness scoring instead of the Glasgow Coma Scale (GCS), as described in Table 1.

**Table 1:** The Physiologic Severity Score (PSS) used for data collection

	0 points	1 point	2 points	3 points	4 points
Respiratory rate	No breathing	<10	> 35	25–35	10–24
Systolic blood pressure	No pulse	< 50	50–69	70–90	> 90
Consciousness	No response	Only to pain	To sound	Confused	Normal

The rated value (0–4 points) for respiratory rate, systolic blood pressure, and consciousness were combined to create the PSS sum score (0–12 points)

The Revised Trauma Score (RTS) uses standard vectors for the three physiological indicators [13]. A Receiver Operating Curve (ROC) analysis of the PSS accuracy in death prediction in the actual study population demonstrated an area under the curve (ROC-AUC) of 0.88 for both the sum of the PSS scores and the weighted PSS ratings. Because no increased predictive value from weighting was observed, sum PSS scores without weighting were used for the statistical analysis. Comparing the PSS ratings on-scene and upon hospital admission, we considered change to a higher score for the PSS-sum (ΔPSS) and each PSS component (ΔRR, ΔBP, and Δconsciousness) to represent positive treatment effects.

### Data collection

The data were collected at two time points. The first point was the first in-field encounter with a paramedic, and the end point was admission to the referral hospital. If data were missing from the hospital admission, the final in-field ratings by the paramedic prior to hospital admission were used as the end point.

The paramedics gathered time factors, tentative clinical diagnoses, PSS scores and life support measures on injury charts in Kurdish and Arabic. Similar vital data were also registered at the endpoints by the emergency room staff. All the data were audited at monthly meetings with the paramedics by the author supervising the program (MKM).

The Injury Severity Score (ISS) was estimated by the senior trauma supervisor (MKM) using the Abbreviated Injury Scale-90 (1998 update), based on the case injury form, as well as hospital information from the surgical reports and X-ray findings. Because of local traditions, autopsy on prehospital fatalities was not performed; in such cases, the ISS values were set conservatively.

### Participant flow

**Fig. 1:**
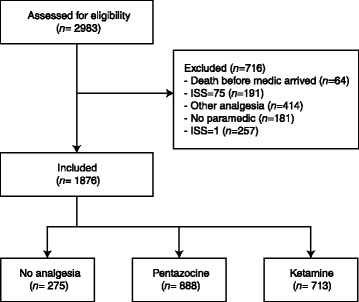
Patient flow chart in this study

A total of 2983 adult patients were registered in the trauma database from January 1997 to December 2006 and assessed for eligibility (Fig. 1). Sixty-four patients who were deceased at the first in-field encounter were excluded from study. A total of 191 patients had an ISS of 75, which was defined as being incompatible with life, and these patients were excluded from the study. Patients with an ISS of 1 were also excluded. An additional 414 patients who received analgesics other than pentazocine and ketamine were excluded. The final study sample included 1876 patients. Some patients were evacuated cross-border to Iran for primary surgery (*n* = 113); endpoint data were unavailable for this patient subsample, and the ISS and PSS were estimated from the prehospital clinical files.

### Subset

Patients with an ISS >8, representing those with moderate and severe injuries, were analyzed as a separate subsamples and in this study were defined as having serious injuries.

### Statistical analysis

Exploratory statistical analyses as well as generalized linear model (GLM) were performed in JMP (SAS Institute, North Carolina, USA) ver. 8 for Mac. Continuous variables are presented as means with 95 % confidence intervals (95 % CI), while the categorical data are expressed as proportions with 95 % CI. Dispersion is shown as median followed by 25–75 % interquartile range. For comparison of means across groups ANOVA was done. If significance was shown in the ANOVA, post hoc Tukey HSD comparison of means were done. Associations between the main outcome variables and potential explanatory factors were analyzed using a GLM linear regression model. The models were fit by forcing the following variables into each model: analgesia (pentazocine vs. no analgesia), analgesia (ketamine vs. pentazocine), gender (male vs. female), age, the time from the injury to the first medical assistance (hours), the time from the injury to the hospital arrival (hours) and the ISS. Standard statistical procedures and normality plots were used to assess the model fit.

### Ethical considerations

The Directorate of Health Suleimaniah, Ministry of Health, Kurdistan region, provided ethical approval for the study (Ref. no. 22082). There is no other authorized committee for medical research ethics in North Iraq. The data were stored in de-identified form and processed according to the Norwegian Social Science Data Service standards (ref. no. 2006/13702).

## Results and discussion

There were more severe injuries in the no analgesia group, as demonstrated by both the anatomic (ISS) and physiologic (PSS) severity scores. Both the in-field response time and total prehospital transit time were longer in the ketamine group. Table 2 shows the baseline description of the three study cohorts.

**Table 2:** Baseline description of the study population

	No analgesia *n* = 275	Pentazocine *n* = 888	Ketamine *n* = 713	Total	Group comparisons
Age (years)	28 (21–39)	30 (22–38)	28 (20–36)	28 (21–37)	*p* = 0.08. No significant differences between groups.
Time from injury to first in-field encounter (hours)	0.5 (0.5–1.0)	0.5 (0.5–0.5)	0.5 (0.5–1)	0.5 (0.5–1)	*p* = 0.02.
					Ketamine vs pentazocine group: *p* = 0.04.
Time from injury to hospital admission (hours)	2 (1.5–3.0)	2 (1.5–3.0)	3 (2.0–4.5)	2.5 (2–3.5)	*p* = <0.0001
					Ketamine vs no analgesia *p* = <0.0001
					Ketamine vs pentazocine *p* = <0.0001
PSS at first in-field encounter	10 (7–11)	10 (4–9)	10 (9–11)	10 (9–11)	*p* = <0.0001
					Pentazocine vs no analgesia *p* = <0.0001
					Ketamine vs no analgesia *p* = <0.0001
Injury severity score (ISS)	5 (4–13)	5 (4–9)	9 (4–9)	5 (4–9)	*p* = <0.0001
					Pentazocine vs no analgesia *p* = <0.0001
					Ketamine vs no analgesia *p* = <0.0001
Males (proportion)	233/275 = 0.85	672/888 = 0.76	602/713 = 0.84	1507/1876 = 0.80	*p* = <0.0001
					All groups significantly different.
Blunt injury (proportion)	196/275 = 0.71	730/888 = 0.82	192/713 = 0.27	1118/1876 = 0.60	*p* = <0.0001
					All groups significantly different.

Continuous values are expressed as median with 25–75 % interquartile range. Proportions are expressed as actual number followed by the calculated proportion

The endpoint scores for each physiological variable as related to the initial physiological score and analgesia are shown in Table 3. The generalized linear model (GLM) showed that analgesia was associated with a significant improvement of PSS (ΔPSS). In the total sample, no difference in ΔPSS between the analgesic groups was observed, but in the subset of patients with ISS >8, ketamine had a significantly better physiological outcome compared to the opioid group (Table 4). No effects of gender, age, time from injury to first medical assistance or time from injury to hospital admission were found. Table 4 also provides details on the confounding effect of injury types on the estimates.

**Table 3:** Change in physiological severity score (PSS) from first point of medical contact to hospital admission, a positive number indicating physiological improvement

	No analgesia *n* = 275	Pentazocine *n* = 888	Ketamine *n* = 713	Group comparisons
Change in PSS sum score	1.3 (1.1–1.5)	1.5 (1.4–1.6)	1.5 (1.4–1.6)	*p* = 0.26.
				No significant differences between means.
Change in respiratory rate score	0.4 (0.3–0.4)	0.5 (0.5–0.6)	0.5 (0.4–0.5)	*p* = 0.003.
				Pentazocine vs no analgesia: *p* = 0.002
				Ketamine vs no analgesia: *p* = 0.02
				Pentazocine vs ketamine: *p* = 0.8
Change in blood pressure score	0.4 (0.3–0.5)	0.6 (0.6–0.7)	0.7 (0.7–0.8)	*p* = <0.0001
				Pentazocine vs no analgesia: *p* = <0.0001
				Ketamine vs no analgesia: *p* = <0.0001
				Ketamine vs pentazocine: *p* = 0.08
Change in consciousness score	0.6 (0.5–0.7)	0.4 (0.3–0.4)	0.3 (0.2–0.3)	*p* = <0.0001
				Pentazocine vs no analgesia: *p* = 0.0006
				Ketamine vs no analgesia: *p* = <0.0001
				Ketamine vs pentazocine: *p* = 0.2

Values are expressed as the mean values with 95 % confidence intervals in brackets

**Table 4:** Variables contributing to the change of PSS sum score in the all-variable generalized linear model

	All		ISS >8	
	Estimate (95 % CI)	*p*	Estimate (95 % CI)	*p*
Intercept	1.02 (0.70–1.34)	<.0001	1.62 (1.03–2.20)	<.0001
Analgesia (pentazocine vs. no analgesia)	0.29 (0.06–0.52)	0.01	0.26 (−0.13–0.65)	0.19
Analgesia (ketamine vs. pentazocine)	−0.04 (−0.24–0.16)	0.68	0.26 (−0.07–0.58)	0.12
Gender (male vs. female)	−0.04 (−0.13–0.06)	0.48	−0.02 (−0.18–0.14)	0.82
Age (years)	−0.01 (−0.01–0.00)	0.10	−0.01 (−0.02–0.00)	0.26
Time from injury to first medical assistance (hours)	0.01 (−0.07–0.08)	0.87	0.00 (−0.13–0.13)	0.99
Time from injury to arrival at the hospital (hours)	0.01 (−0.03–0.04)	0.59	0.00 (−0.06–0.05)	0.96
Injury severity score (ISS)	0.04 (0.03–0.06)	<.0001	0.01 (−0.01–0.03)	0.20
Blunt type of injury	0.00 (−0.09–0.10)	0.97	−0.09 (−0.25–0.06)	0.24

### Respiratory rate

The GLM analysis showed that analgesia was associated with a positive effect on respiratory rate score, while no difference was found between pentazocine and ketamine (Table 5). Increasing in-field response time was associated with a worse respiratory rate, while increasing total prehospital time was associated with a better respiratory rate. The model also gives estimates on the confounding effect of injury types on the estimates and shows that having an extremity injury was associated with deteriorating respiratory rate score.

**Table 5:** Variables contributing to the physiologic score changes in the all-variable generalized linear models

	All		ISS >8 subgroup	
	Estimate (95 % CI)	*p*	Estimate (95 % CI)	*p*
Variables contributing to respiratory rate score changes				
Intercept	0.21 (0.08–0.35)	0.002	0.50 (0.25–0.75)	<.0001
Analgesia (pentazocine vs. no analgesia)	0.20 (0.10–0.30)	<.0001	0.21 (0.04–0.38)	0.015
Analgesia (ketamine vs. pentazocine)	−0.04 (−0.12–0.04)	0.35	0.01 (−0.13–0.15)	0.88
Gender (male vs. female)	−0.03 (−0.07–0.02)	0.21	0.00 (−0.07–0.07)	0.98
Age (years)	0.00 (0.00–0.00)	0.86	0.00 (−0.01–0.00)	0.50
Time from injury to first medical assistance (hours)	−0.03 (−0.07–0.00)	0.04	−0.04 (−0.09–0.02)	0.21
Time from injury to arrival at the hospital (hours)	0.02 (0.00–0.03)	0.02	0.01 (−0.02–0.03)	0.53
Injury severity score (ISS)	0.01 (0.01–0.02)	<.0001	0.00 (−0.01–0.01)	0.97
Blunt type of injury	0.00 (−0.04–0.04)	0.95	−0.02 (−0.09–0.05)	0.55
Variables contributing to the change in the blood pressure rated score				
Intercept	0.38 (0.23–0.53)	<.0001	0.60 (0.33–0.86)	<.0001
Analgesia (pentazocine vs. no analgesia)	0.28 (0.17–0.38)	<.0001	0.23 (0.06–0.41)	0.01
Analgesia (ketamine vs. pentazocine)	0.03 (−0.06–0.12)	0.52	0.16 (0.02–0.31)	0.03
Gender (male vs. female)	−0.01 (−0.06–0.03)	0.60	−0.01 (−0.08–0.07)	0.89
Age (years)	0.00 (−0.01–0.00)	0.03	0.00 (−0.01–0.00)	0.32
Time from injury to first medical assistance (hours)	0.03 (0.00–0.07)	0.08	0.02 (−0.03–0.08)	0.44
Time from injury to arrival at the hospital (hours)	−0.01 (−0.03–0.01)	0.20	0.00 (−0.03–0.02)	0.80
Injury severity score (ISS)	0.02 (0.01–0.02)	<.0001	0.00 (−0.01–0.01)	0.78
Blunt type of injury	−0.05 (−0.09–0.00)	0.03	−0.08 (−0.15 – −0.01)	0.03
Variables contributing to consciousness rated score changes				
Intercept	0.43 (0.28–0.59)	<.0001	0.52 (0.24–0.80)	0.0003
Analgesia (pentazocine vs. no analgesia)	−0.18 (−0.29– −0.07)	0.001	−0.18 (−0.37–0.00)	0.06
Analgesia (ketamine vs. pentazocine)	−0.03 (−0.13–0.06)	0.51	0.08 (−0.07–0.24)	0.29
Gender (male vs. female)	0.00 (−0.04–0.05)	0.88	−0.01 (−0.09–0.06)	0.71
Age (years)	0.00 (−0.01–0.00)	0.14	0.00 (−0.01–0.00)	0.41
Time from injury to first medical assistance (hours)	0.01 (−0.03–0.04)	0.64	0.01 (−0.05–0.07)	0.71
Time from injury to arrival at the hospital (hours)	0.00 (−0.01–0.02)	0.77	−0.01 (−0.03–0.02)	0.67
Injury severity score (ISS)	0.02 (0.01–0.02)	<.0001	0.01 (0.00–0.02)	0.01
Blunt type of injury	0.05 (0.00–0.09)	0.04	0.01 (−0.07–0.08)	0.88

### Blood pressure

Receiving analgesia was associated with a better blood pressure score. In the subset of patients with serious injuries, ketamine analgesia was associated with a significant positive change in systolic blood pressure.

### Consciousness

Analgesia had a negative impact on the level of consciousness. No significant difference between the drugs was observed.

## Discussion

The study shows that prehospital analgesia was associated with an improvement of physiological severity indicators. For patients with an ISS >8, ketamine analgesia was associated with a better treatment effect than opioid analgesia. Better treatment effects on respiration and blood pressure contributed to the better physiologic outcome.

### Limitations

First, the study was strictly observational, and treatment was decided by paramedic preference; therefore, some baseline characteristics varied between the three subsets. The patients not receiving analgesia were more severely injured. Further, the in-field response times and total transit times were higher in the ketamine cohort. Ketamine was regularly used in penetrating injuries, most of which were land mine injuries that occurred in remote rural areas, while pentazocine often was the analgesic of choice in blunt trauma cases, which were predominantly caused by road traffic accidents. The confounder effects of these variables were adjusted for in the GLM model.

Second, a small subset of patients was given diazepam (*n* = 228), particularly when the evacuations were risky and agitation might have caused security problems. Diazepam may have affected the outcome indicators by depressing the respiratory rates, blood pressure, and consciousness.

Third, there were shortcomings with the study protocol. The actual study used data from a trauma registry that was designed with the main aim to monitor trauma mortality. The registry did not include data on dosages and frequency on in-field drug administration. It was possible to write free text on the injury charts, but the side effects of analgesics were not systematically registered. However, the senior trauma system supervisor (MKM) systematically audited the treatments and outcomes for all of the study patients at monthly meetings.

### Interpretation

#### Airway control, respiration and circulation

The study indicates that prehospital analgesia has a positive effect on systolic blood pressure. Although it may appear that this effect was confounded by volume replacement, the fluid protocol was similar for all three cohorts. In patients with severe injuries, ketamine had a significantly better effect on blood pressure compared to opioid analgesia. This finding corresponds well with a small Swedish randomized study that demonstrated that the addition of ketamine to opioid analgesia increases blood pressure after trauma [10].

The increased blood pressure for seriously injured patients in the ketamine group is a clinically important finding. The optimal blood pressure target for trauma victims is controversial, and it is not possible to determine a general blood pressure target in the current literature [14]. In this study, the elevation of blood pressure to 90 mmHg was considered optimal.

Another clinically important finding in this study was the improved respiratory rates in patients receiving analgesia. Pain relief may normalize the respiratory rate, increase the ventilated parts of the lungs and assist in oxygenation.

Airway control is crucial, particularly with mass casualties in chaotic settings in which trauma victims cannot be monitored closely on-scene and during evacuation. The London helicopter emergency medical service has reported emergency physicians administering ketamine to 1030 adult patients, and no significant airway complications were observed. Ketamine has been used in this service by a physician-paramedic team to provide analgesia, sedation and (rarely) the induction of anesthesia [8].

Another study described ketamine use in 40 patients in a physician-equipped regional aeromedical critical care service. The patients maintained airway responsiveness and adequate oxygen saturations [15]. This study concluded that ketamine was an ideal drug for use in many prehospital situations. This experience demonstrated that ketamine was safe and effective, and it may be more appropriate than other drugs currently used by prehospital providers.

#### Safety and adverse events

We have limited adverse event data in our study population, as only major side effects would affect physiologic variables. There is a free text field in the injury chart, and one of the authors (MKM) conducted monthly meetings with the paramedics in an attempt to collect this information.

This study was performed in a low-resource setting, but even in high-income countries the close monitoring of patients in the prehospital setting is challenging. In the study setting, it is of utmost importance that the drugs given are safe and do not cause cardiorespiratory trouble.

Adverse events associated with ketamine administration were studied in a narrative review of 88 studies. These studies reported adverse events for more than 70,000 patients; a cardiorespiratory event of lasting significance reported (hypoxic cardiac arrest) was recorded in one patient [2].

Another concern is the respiratory depression caused by opioids [16], while ketamine seldom causes respiratory depression. Our study was performed in a setting where evacuations were commonly conducted with few resources and poor monitoring, the injury scene was chaotic, the transport was long and dangerous, and mass casualties were often treated with limited numbers of health personnel. This situation makes close patient monitoring challenging. In the London study of prehospital ketamine, only 6/1030 patients experienced decreased oxygen saturation from the initial saturation to the saturation at admittance, and the most pronounced change was a decrease to a saturation of 89 %. No significant complications or airway complications were observed in this study [8]. A definite advantage of ketamine for disorganized scenes is that ketamine has been shown to be safe, even in dosages higher than that required for analgesia [3].

#### Traumatic brain injuries

In our study, we could observe that receiving analgesia was associated with decreased consciousness. It is expected that analgesics with a central mechanism of action might influence consciousness. Similar findings have been used as an argument for not providing pain relief to patients with suspected brain injuries. It is proposed that pain relief creates problems in assessing consciousness and the neurologic findings in traumatic brain injury cases.

Current recommendations for the prehospital care of patients with head injuries emphasize preventing secondary brain injuries by avoiding hypoxemia and hypotension [17]. Our study indicated that ketamine contributed to increased systolic blood pressure in trauma victims, which may contribute to increased cerebral perfusion.

Ketamine administration has been controversial in traumatic brain injuries, also because some early studies have reported increased intracranial pressure (ICP) in patients receiving ketamine [18, 19]. For this reason, ketamine was not used in patients with traumatic brain injuries or for traffic accidents in this study. Later studies have reported conflicting findings [20–22]. This discrepancy was discussed in a meta-analysis that refuted the contraindications in using ketamine in neurologically impacted patients. The study concluded that there was no increase in intracranial pressure and provided evidence that hemodynamic stimulation may improve cerebral perfusion [23]. One might argue that increased cerebral perfusion pressure resulting from increased systolic blood pressure can be of greater importance than merely creating difficulty for later assessment in the emergency room.

## Conclusion

Prehospital analgesia for trauma victims improves physiologic severity indicators in a low-resource trauma system. Compared to pentazocine, ketamine was associated with improved blood pressure for patients with serious injuries. Ketamine seems to be a good choice for prehospital analgesia in trauma victims, particularly in a low-resource setting.
